# Isolation and identification of antibiotic-resistant bacteria in hospital tap water

**DOI:** 10.3389/frabi.2026.1901158

**Published:** 2026-09-15

**Authors:** Wasan Mohammed Abdulzahra, Zainab Qasim Hilo, Aswan Mahmood Hashim, Haider Nadhom Athab, Kawthar Abd Al-Hadi Abd Al-Karim, Hawraa Majid Hassan, Hussein Fadil Ibrahim, Ayad M. J. Al-Mamoori

**Affiliations:** 1 University of Babylon, College of Basic Education, Hillah, Babil, Iraq; 2 DNA Research Center, University of Babylon, Hillah, Babil, Iraq; 3 Independent Researcher, Hillah, Babil, Iraq; 4 University of Babylon, College of Science, Biology Department, Hillah, Babil, Iraq

**Keywords:** antibiotic resistance, bacterial species, hospital tap water, infection control, water safety, premise plumbing, VITEK 2

## Abstract

**Introduction:**

Hospital tap water can act as a reservoir of opportunistic and potentially antibiotic-resistant bacteria, yet baseline data on the bacterial communities and antimicrobial resistance profiles of premise-plumbing systems remain scarce in Iraq. This study investigated the bacterial species present in hospital tap water and their antibiotic resistance profiles, with the aim of supporting infection control measures in healthcare settings.

**Methods:**

Tap water samples were collected from three hospitals in Babil Governorate, Iraq (Merjan Teaching Hospital, Turkish Hospital, and Al-Jiumhori Hospital) and were processed for heterotrophic plate counts and bacterial isolation. Isolates were identified to the species level using the VITEK 2 Compact automated phenotypic system, and antimicrobial susceptibility was determined on the same platform and interpreted according to Clinical and Laboratory Standards Institute guidelines.

**Results:**

A total of 11 bacterial isolates were recovered. The highest bacterial load was recorded in the Turkish Hospital sample (3.43 × 10^4^ CFU/mL). The identified species comprised Granulicatella elegans, *Acinetobacter haemolyticus, Pseudomonas alcaligenes, Moraxella lacunata, Francisella tularensis, Aeromonas sobria, Pseudomonas stutzeri*, and *Sphingomonas paucimobilis*. The single Gram-positive isolate, G. elegans, showed the highest resistance (10 of 14 agents tested; 71.4%), whereas the Gram-negative isolates were largely susceptible, with only sporadic resistance detected in *P. stutzeri* and *M. lacunata*.

**Discussion:**

Because several species were represented by a single isolate and identification relied on an automated phenotypic platform, these resistance estimates are descriptive and should be interpreted with caution. The findings nonetheless confirm the presence of opportunistic and potentially resistant bacteria in hospital tap water and underscore the need for routine microbiological monitoring and improved water safety protocols.

## 1 Introduction

Since water is necessary for life, improving the availability of clean drinking water can have significant positive health consequences. Everyone must have access to a sufficient, safe, and useful supply. To ensure that the drinking water is as safe as possible, every effort should be done (WHO, 2008). A distribution of Gram-negative and non-fermentative bacteria found in hospital tap water sources was presented to warn physicians about potential environmental and medical device contamination or to educate patients about risky behaviors (Vincenti et al, 2014) In order to determine the quantity and kinds of bacteria present in three various water sources, including bottled water, water cooler water, and tap water, many different types of water were gathered and examined for heterotrophic plate count bacteria (*Pseudomonas* and total coliforms). The virulence traits were identified for every isolate, and antibiotic susceptibility testing was carried out (Edberg et al, 1996). Multiple antibiotic-resistant bacteria that are resistant to two or more antibiotics were detected in water samples from seven towns. The standard plate count bacteria were found at frequencies of 20.4% and 18.6% in two distinct raw water supplies for two towns, while 36.7% and 67.8% of the standard plate count populations from sites inside related distribution systems were multiple-antibiotic resistant. The multiple-antibiotic-resistant Gram-negative, non-fermentative rods and multiple-antibiotic-resistant Gram-positive cocci were found to be common in treated waters more often than in sources of untreated waters, which included *Acinetobacter*, *Moraxella*-like group M, *Pseudomonas*, and *Alcaligenes*. The differences from site to site in antibiotic-resistant organism occurrences and generic types imply that bacterial populations in tap water may have been impacted by the shedding of the bacteria into the pipelines (Armstrong et al, 1981). Water is essential to life, but many people lack access to clean drinking water, and lots of people die from bacterial illnesses that are spread by contaminated water. The importance of emerging and dangerous disease strains that are transmitted by drinking water and the analysis of water based on fecal indicator bacteria, which examines how these bacteria exist in the feces of both humans and animals and are carried by both hosts and the environment (Cabral, 2010), should be considered. Within the family Carnobacteriaceae, the genus *Granulicatella* comprises bacteria that are found in the urogenital tract, gastrointestinal tract, and upper respiratory tract (Procop et al., 2020). In rare instances, it leads to human illness (Schwede et al, 2007). *G. elegans* is known as *Abiotrophia elegans* and initially identified since 1998 as a facultative anaerobic Gram-positive bacteria that was non-motile, oxidase-negative, and catalase-negative (Cargill et al, 2012). Nevertheless, the bacterium may show pleomorphism and variability when stained with Gram stain: cocci in nutrient-rich media and bacilli in nutrient-depleted media are examples of different morphologies (Roggenkamp et al., 1998). Thus, *Granulicatella* species, which were once known as nutritionally variable streptococci, can cause endocarditis and other diseases (Giuliano et al., 2012). Compared to endocarditis produced by streptococci, endocarditis induced by these organisms is more acute and has a higher relapse rate, a longer cycle of illness, and a higher fatality rate (Padmaja et al., 2014), with side effects such as embolization, abscess formation, and heart failure (Adam et al., 2015). *Acinetobacter* spp., common environmental bacteria, were isolated from the connected tap water and the municipal drinking water treatment facility. These were identified at the species level (Narciso-da-Rocha et al., 2013). When exposed to arid conditions, Gram-negative bacteria rarely survive in the environment; nevertheless, *Acinetobacter* species can do so and cause nosocomial illnesses (Jawad et al., 1994). Infections in clinical settings are frequently linked to *Acinetobacter* spp. Rarely are reports of infections brought on by environmental isolates (Govender et al., 2020). *Pseudomonas alcaligenes*, also known as strain MRCP1333T, is a rod-shaped, aerobic, Gram-negative bacteria that does not create endospores, and it was isolated from a Japanese hospital patient’s feces. At temperatures between 15 °C and 40 °C (ideally between 25 °C and 35 °C), MRCP1333T grew (Ono et al., 2023). Antibiotic resistance is a known feature of *Pseudomonas* that includes pathogenic and environmental species. It can acquire genes of resistance from enteric bacteria that are discharged through feces into the environment. These fecal bacteria have come into touch with populations of *Pseudomonas* (Camiade et al., 2020). The main similarity between the two very different bacterial genera *Moraxella* and *Acinetobacter* is that their representatives may appear identical when stained with Gram stain. *Acinetobacter* species make up between 1% and 3% of the non-fastidious Gram-negative bacilli isolated in a typical hospital laboratory. Free-living microbes called *Acinetobacter* species are frequently found in soil and water. The majority of *Acinetobacter* laboratory isolates are not linked to serious infections (Rosenthal, 2020). In addition to exposure to infected animals, water, and aerosols, *Francisella tularensis* can spread by arthropod bites (Aghamohammad et al., 2022). The gamma subclass of proteobacteria includes *Francisella* that is the member of the Francisellaceae family. The genus includes unclassified *Francisella* species, and *Francisella tularensis*, *Francisella novicida*, *Francisella philomiragia*, and *Francisella noatunensis* are among them. There are differences between *Francisella* species and *F. tularensis*, *F*. *novicida*, and *F. philomiragia*. The Gram-negative coccobacilli that make up the genus *Francisella* can be identified by a number of characteristics (Petersen et al., 2011). A common non-fluorescent denitrifying bacteria in the environment, *Pseudomonas stutzeri* has also been identified as an opportunistic pathogen in humans (Lalucat et al., 2006). *Aeromonas* spp. exist everywhere in aquatic environments, and it can cause diseases (Borella et al., 2020). Numerous aquatic settings naturally harbor the genus *Aeromonas*, which consists of over 30 Gram-negative bacterial species, mostly opportunistic germs (Pessoa et al., 2019). *Aeromonas* sp. is reported in watery ecosystems and is a pathogen in humans and animals (Silva et al., 2022). The bacterium genus *Aeromonas*, which is extensively dispersed and important for food, animal, and human diseases, is found in aquatic settings. The environmental isolates of this genus often have acquired resistance traits, which are used as indicators to evaluate the water quality (Grilo et al., 2020). The use of water in a hospital can lead to the growth of *Sphingomonas paucimobilis*, a Gram-negative, aerobic, non-fermentative opportunistic bacillus (Menekşe et al., 2023). Previously, it was a rare opportunistic infection found in clinical specimens (Rohilla et al., 2021). It is a newly discovered aerobic, non-fermenting, Gram-negative, opportunistic bacterium that causes a lot of infections linked to healthcare. It may be another new source of healthcare-associated infections that spread quickly, which emphasizes the need for strict monitoring and control measures (Bavaro et al., 2020).

Despite the recognized importance of premise-plumbing microbiology, baseline data on the bacterial communities and antimicrobial resistance profiles of hospital tap water remain scarce in Iraq. The present study was therefore designed to address the following research questions: Which culturable bacterial species are present in the tap water of selected hospitals in Babil Governorate, and what are their antibiotic susceptibility patterns? We hypothesized that hospital tap water in this setting harbors opportunistic, non-fermentative Gram-negative bacteria together with occasional Gram-positive organisms and that a subset of these isolates would display reduced susceptibility to clinically relevant antibiotics. Accordingly, the specific objectives were (i) to enumerate the heterotrophic bacterial load in tap water from three hospitals, (ii) to isolate and identify the recovered bacteria to the species level, and (iii) to determine their antimicrobial susceptibility profiles in order to provide a baseline for water safety and infection control planning.

## 2 Materials and methods

### 2.1 Sampling

Tap water samples were collected from three public hospitals in Babil Governorate, Iraq: Merjan Teaching Hospital, Turkish Hospital, and Al-Jiumhori Hospital. From each hospital, one composite tap water sample was obtained from a point-of-use cold-water tap (a hand-washing basin) located in an inpatient patient-care area, giving three hospital samples in total; for the heterotrophic plate count, each composite sample was subsequently processed in triplicate. Sampling was carried out during the morning of routine working days in the summer of 2023, when ambient temperatures ranged between approximately 35 °C and 45 °C. To reflect the microbial load to which patients and healthcare workers are realistically exposed at the point of use, samples were collected as first-draw water without prior flushing of the tap, after the external surface of the outlet had been wiped and briefly flamed, with any fitted aerator left in place. A volume of 500 mL was collected from each tap into sterile borosilicate-glass bottles containing sodium thiosulfate (final concentration, 18 mg/L) to neutralize any residual free chlorine. The bottles were labeled, transported to the laboratory in an insulated cool-box at approximately 4 °C, and processed within 2 h of collection. All sampling and downstream handling were performed using aseptic technique to minimize the risk of external contamination.

### 2.2 Total plate count of bacteria

After thoroughly mixing all of the samples, 1 mL of the tap water sample was added to 9 mL of normal saline (diluent) (stock 10^-1^) to create three serial dilutions (10^–2^ and 10^-3^). Using a disposable spreader, 10 µL of the final dilution was spread onto a nutrient agar plate. The plates were incubated inverted at 37 °C for 24 h; to improve accuracy, each sample was examined in triplicate, and only plates bearing 30–300 colonies were selected for counting (Goldman and Green, 2008).

### 2.3 Isolation and identification of bacterial species

Immediately after collection, the total heterotrophic plate count was determined as described above, and morphologically distinct colonies were selected from the countable plates for further characterization. To obtain pure cultures, each selected colony was sub-cultured by streaking onto fresh nutrient agar and incubated at 37 °C for 24 h; this purification step was repeated until a uniform colony morphology was obtained, and purity was confirmed by Gram staining. Representative purified colonies, rather than every colony present, were carried forward for identification, and the number of isolates retained per hospital reflected the number of distinct colony morphotypes recovered from each sample. Each pure isolate was Gram-stained to record its Gram reaction and was then identified to the species level using the VITEK 2 (bioMérieux, France) automated system with the appropriate identification card, following the manufacturer’s instructions (Cheesbrough, 1991). It should be noted that VITEK 2 provides biochemical (phenotypic) identification; molecular confirmation of the isolates was not performed in the present study.

### 2.4 Antibacterial susceptibility testing

The antibiotic susceptibility of the isolated bacterial species was determined using the automated VITEK 2 Compact system (bioMérieux, France). Following the manufacturer’s protocols, bacterial suspensions were prepared to a standardized McFarland turbidity. These suspensions were then inoculated into the appropriate VITEK 2 AST (antimicrobial susceptibility testing) cards. The cards were incubated and automatically read by the instrument, which analyzes microbial growth in the presence of various antibiotics to determine the minimum inhibitory concentrations (MICs). The results were interpreted according to the Clinical and Laboratory Standards Institute (CLSI) guidelines to classify the isolates as susceptible, intermediate, or resistant. Each isolate was tested on a single VITEK 2 AST card; antimicrobial susceptibility testing was therefore performed as one determination per isolate rather than in replicate, and the panel of antibiotics reported by the instrument differed between Gram-positive and Gram-negative organisms.

### 2.5 Quality control and contamination prevention

Standard quality control and quality assurance measures were applied throughout the study. The sterility of all culture media and diluents was verified by incubating representative uninoculated batches at 37°C for 24–48 h before use, and only batches that showed no growth were used. All manipulations were performed using aseptic technique beside a flame; a negative process control consisting of sterile normal saline was carried through the dilution and plating steps during each sampling run, and uninoculated control plates were exposed during processing to monitor for environmental cross-contamination. Reference strains were used to control the performance of susceptibility testing: *Escherichia coli* ATCC 25922 and *Staphylococcus aureus* ATCC 29213 served as the antimicrobial susceptibility quality-control strains, and AST runs were accepted only when the control values fell within the CLSI-defined acceptable ranges. VITEK 2 identification and AST results were accepted only when the instrument returned an acceptable confidence level and completed the analysis without critical error flags; any isolate failing these acceptance criteria was re-tested from a fresh pure subculture.

### 2.6 Statistical analysis

Antimicrobial susceptibility data were analyzed using IBM SPSS Statistics version 32.0.0, and figures were prepared using GraphPad Prism version 11.0.2. Resistance rates were calculated as R/(S+I+R), and non-susceptibility rates as (I+R)/(S+I+R). Two-sided 95% confidence intervals were calculated by the Wilson score method for every proportion reported, and categorical comparisons were made using Fisher's exact test, with Bonferroni adjustment for multiple pairwise comparisons, with *p* < 0.05 considered statistically significant.

## 3 Results

### 3.1 Total plate count for bacteria

The total plate count analysis revealed bacterial contamination in the tap water samples from all three hospitals surveyed. The highest bacterial load was observed in the sample from Turkish Hospital, with a count of 3.43 × 10^4^ CFU/mL, while the samples from Merjan Teaching Hospital and Al-Jiumhori Hospital showed lower counts of 2.70 × 10³ and 1.32 × 10³ CFU/mL, respectively. When interpreted against drinking water quality benchmarks, all three values greatly exceeded the heterotrophic plate count level of ≤500 CFU/mL that is commonly applied as an indicator of acceptable treated water quality, and the recovery of culturable bacteria is inconsistent with the World Health Organization guideline that fecal indicator organisms should be absent from 100 mL of water intended for human consumption (WHO, 2008). The observed counts likewise exceeded the microbiological limits set by the Iraqi national drinking water standard. Taken together, these results indicate a microbiological water quality failure across all three hospitals and a potential exposure risk that is of particular concern for immunocompromised patients and other vulnerable groups.

There are 11 bacterial isolates in the raw inventory (**Table 1**). Merjan Teaching Hospital provided three isolates and Turkish Hospital and Al-Jiumhori Hospital provided four isolates each. One isolate was Gram-positive, while the other 10 isolates were Gram-negative.

**Figure 1** presents these heterotrophic plate counts on a logarithmic scale (log^10^ CFU/mL) for the three hospital samples. The values summarize the plate count readings obtained for each composite sample and, because only one composite sample was examined per hospital, they should not be interpreted as independent between-site replicates.

**Figure 1:**
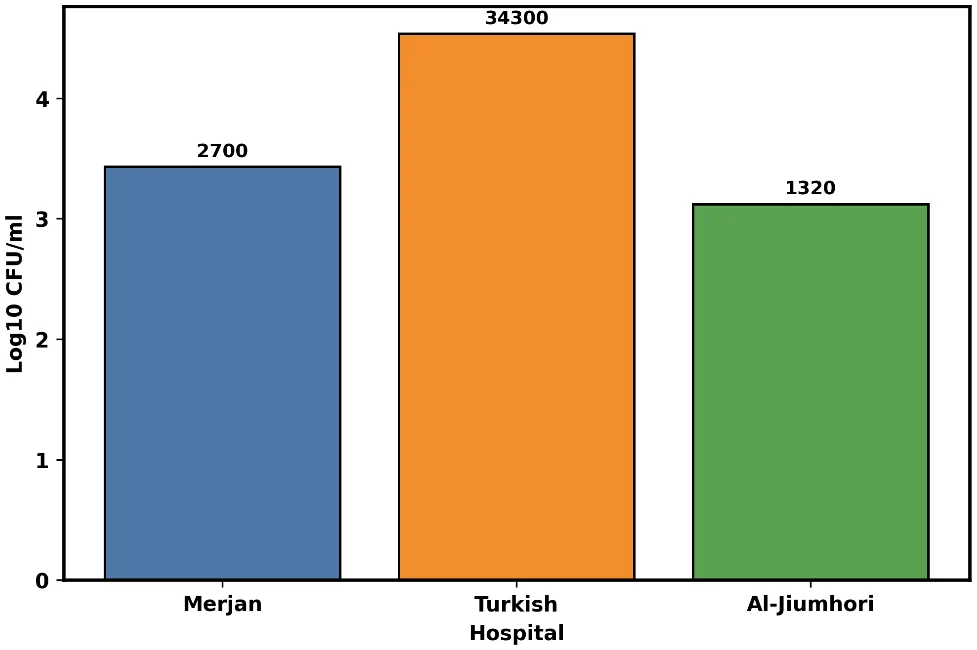
Total plate count of bacteria among hospital tap water samples.

### 3.2 Bacterial isolates

The Gram-stain results showed that one isolate was Gram-positive and 10 were Gram-negative. Subsequent identification using VITEK 2 assigned the recovered isolates to the following species: *Granulicatella elegans*, *Acinetobacter haemolyticus*, *Pseudomonas alcaligenes*, *Moraxella lacunata*, *Francisella tularensis*, *Aeromonas sobria*, *Pseudomonas stutzeri*, and *Sphingomonas paucimobilis*.

Most species were represented by one isolate only, as shown in the species distribution. Species-level percentages are therefore only descriptive, as there were two isolates of each of the following species: *Aeromonas sobria*, *Pseudomonas stutzeri*, and *Sphingomonas paucimobilis*.

The number and composition of the isolates retrieved from each hospital source is summarized in the stacked vertical column chart. Three isolates were from Merjan Teaching Hospital and four each were from Turkish Hospital and Al-Jiumhori Hospital. The majority of species were sampled once except that *Aeromonas sobria*, *Pseudomonas stutzeri*, and *Sphingomonas paucimobilis* which were sampled two times in the data set (**Figure 2**).

**Figure 2:**
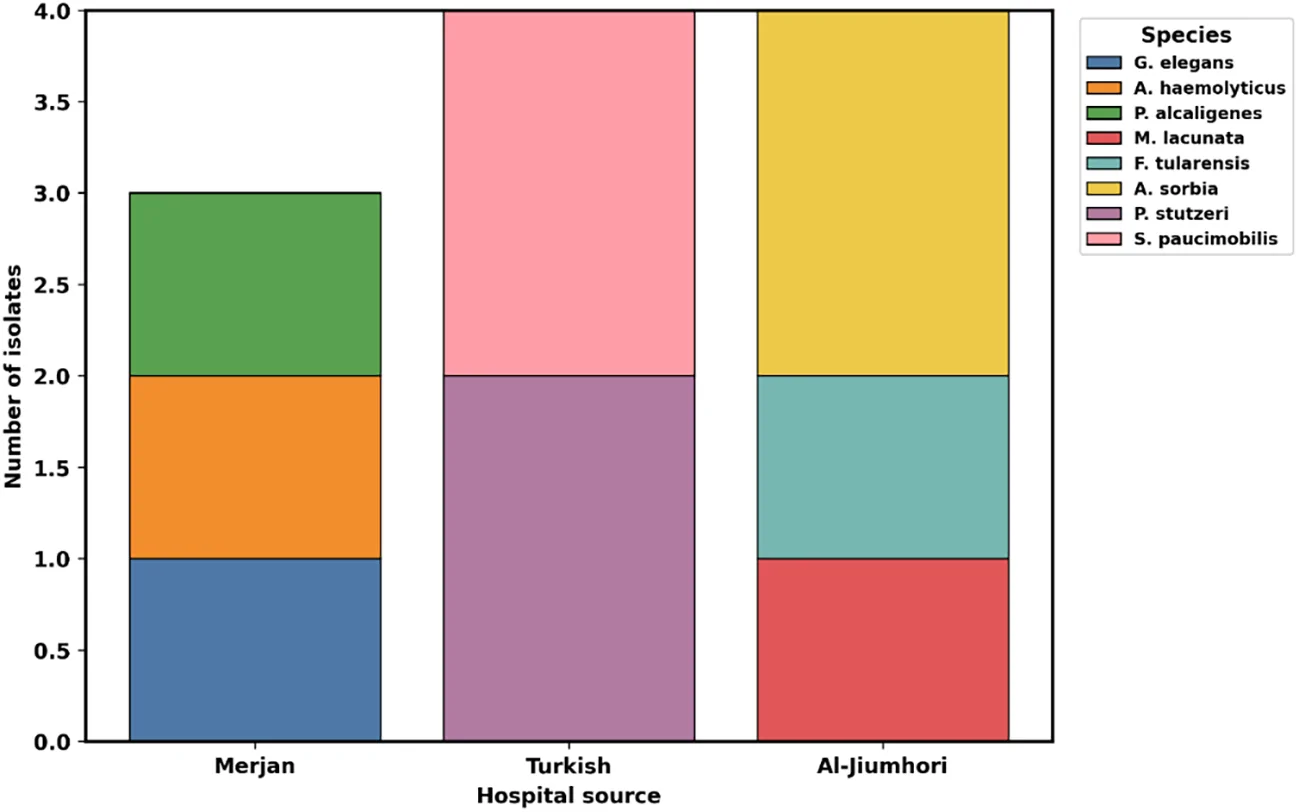
Distribution of bacterial isolates according to hospital source.

The tap water of all three hospitals carried a high bacterial load: Turkish Hospital had the highest count (3.43 × 10^4^ CFU/mL), Al-Jiumhori Hospital had the lowest (1.32 × 10^3^ CFU/mL), and Merjan Teaching Hospital had an intermediate value (2.70 × 10^3^ CFU/mL). **Table 1** lists the raw isolate inventory, and **Table 2** shows the corresponding species distribution across the 11 isolates. Merjan Teaching Hospital yielded three isolates (*Granulicatella elegans*, *Acinetobacter haemolyticus*, and *Pseudomonas alcaligenes*), Turkish Hospital yielded four isolates belonging to two species (*Pseudomonas stutzeri* and *Sphingomonas paucimobilis*), and Al-Jiumhori Hospital yielded four isolates belonging to three species (*Moraxella lacunata*, *Francisella tularensis*, and *Aeromonas sobria*). The relatively high bacterial counts and species diversity are most plausibly attributable to the contamination of the premise-plumbing system, for example, through biofilm formation in aging, corroded, or damaged pipework and taps, inadequate disinfection, and the heavy human traffic characteristic of hospital environments.

**Table 1:** Raw isolate inventory according to hospital source and Gram reaction.

Isolate ID	Species	Gram reaction	Source hospital	Replicate
ISO-01	*Granulicatella elegans*	Gram-positive	Merjan Teaching Hospital	1 of 1
ISO-02	*Acinetobacter haemolyticus*	Gram-negative	Merjan Teaching Hospital	1 of 1
ISO-03	*Pseudomonas alcaligenes*	Gram-negative	Merjan Teaching Hospital	1 of 1
ISO-04	*Moraxella lacunata*	Gram-negative	Al-Jiumhori Hospital	1 of 1
ISO-05	*Francisella tularensis*	Gram-negative	Al-Jiumhori Hospital	1 of 1
ISO-06	*Aeromonas sobria*	Gram-negative	Al-Jiumhori Hospital	1 of 2
ISO-07	*Aeromonas sobria*	Gram-negative	Al-Jiumhori Hospital	2 of 2
ISO-08	*Pseudomonas stutzeri*	Gram-negative	Turkish Hospital	1 of 2
ISO-09	*Pseudomonas stutzeri*	Gram-negative	Turkish Hospital	2 of 2
ISO-10	*Sphingomonas paucimobilis*	Gram-negative	Turkish Hospital	1 of 2
ISO-11	*Sphingomonas paucimobilis*	Gram-negative	Turkish Hospital	2 of 2

**Table 2:** Distribution of bacterial species according to hospital source.

Species	Merjan teaching hospital	Turkish hospital	Al-Jiumhori hospital	Total isolates	Percentage of total isolates (%)
*Granulicatella elegans*	1	0	0	1	9.1
*Acinetobacter haemolyticus*	1	0	0	1	9.1
*Pseudomonas alcaligenes*	1	0	0	1	9.1
*Moraxella lacunata*	0	0	1	1	9.1
*Francisella tularensis*	0	0	1	1	9.1
*Aeromonas sobria*	0	0	2	2	18.2
*Pseudomonas stutzeri*	0	2	0	2	18.2
*Sphingomonas paucimobilis*	0	2	0	2	18.2

### 3.3 Antibiotic susceptibility assay results

#### 3.3.1 Antibiotic susceptibility for Gram-positive bacteria

The results revealed that *Granulicatella elegans* is resistant to benzylpenicillin, oxacillin, gentamicin, ciprofloxacin, moxifloxacin, erythromycin, clindamycin, teicoplanin, tetracycline, and rifampicin while sensitive to tigecycline, fusidic acid, and trimethoprim/sulfamethoxazole and intermediate to vancomycin; the recalculated resistance rate (10 resistant out of 14 agents tested) is therefore 71.4%.

*Granulicatella elegans* had the highest resistance, with 10 resistant out of the 14 tested antibiotics. *Pseudomonas stutzeri* ISO-09 was resistant to piperacillin, piperacillin/tazobactam, and cefepime. One result of colistin resistance was obtained for *Moraxella lacunata* and *Sphingomonas paucimobilis* ISO-10. Although no resistant results were recorded for the remaining isolates, these zero resistance rates must be interpreted with caution, because resistance determinants were not investigated at the molecular level and not all antibiotics were tested against every isolate.

**Table 3** shows that *Granulicatella elegans* was resistant to most of the agents tested. This pattern is consistent with the intrinsically reduced susceptibility of this fastidious, nutritionally variant Gram-positive organism and with the thick peptidoglycan cell wall that restricts the access of several agent classes, although the isolate remained susceptible to tigecycline, fusidic acid, and trimethoprim/sulfamethoxazole.

**Table 3:** Recalculated isolate-level antimicrobial resistance profile from raw AST results.

Isolate ID	Species	Gram reaction	Source hospital	No. of tested results	S	I	R	Resistance rate (%)	95% CI	Non-susceptible rate (%)
ISO-01	*Granulicatella elegans*	Gram-positive	Merjan Teaching Hospital	14	3	1	10	71.4	45.4–88.3	78.6
ISO-02	*Acinetobacter haemolyticus*	Gram-negative	Merjan Teaching Hospital	15	15	0	0	0.0	0.0–20.4	0.0
ISO-03	*Pseudomonas alcaligenes*	Gram-negative	Merjan Teaching Hospital	14	14	0	0	0.0	0.0–21.5	0.0
ISO-04	*Moraxella lacunata*	Gram-negative	Al-Jiumhori Hospital	16	14	1	1	6.2	1.1–28.3	12.5
ISO-05	*Francisella tularensis*	Gram-negative	Al-Jiumhori Hospital	4	3	1	0	0.0	0.0–49.0	25.0
ISO-06	*Aeromonas sobria*	Gram-negative	Al-Jiumhori Hospital	10	10	0	0	0.0	0.0–27.8	0.0
ISO-07	*Aeromonas sobria*	Gram-negative	Al-Jiumhori Hospital	10	10	0	0	0.0	0.0–27.8	0.0
ISO-08	*Pseudomonas stutzeri*	Gram-negative	Turkish Hospital	14	13	1	0	0.0	0.0–21.5	7.1
ISO-09	*Pseudomonas stutzeri*	Gram-negative	Turkish Hospital	14	11	0	3	21.4	7.6–47.6	21.4
ISO-10	*Sphingomonas paucimobilis*	Gram-negative	Turkish Hospital	16	14	1	1	6.2	1.1–28.3	12.5
ISO-11	*Sphingomonas paucimobilis*	Gram-negative	Turkish Hospital	16	16	0	0	0.0	0.0–19.4	0.0

The heatmap in **Figure 3** presents a tabular overview of susceptible, intermediate, and resistant results for the available isolate–antibiotic combinations. The coordinated color scheme makes it easier to follow the resistance patterns and highlights that resistant results were largely limited to *Granulicatella elegans*, with isolated resistance results for *Moraxella lacunata*, *Pseudomonas stutzeri*, and one isolate of *Sphingomonas paucimobilis*.

**Figure 3:**
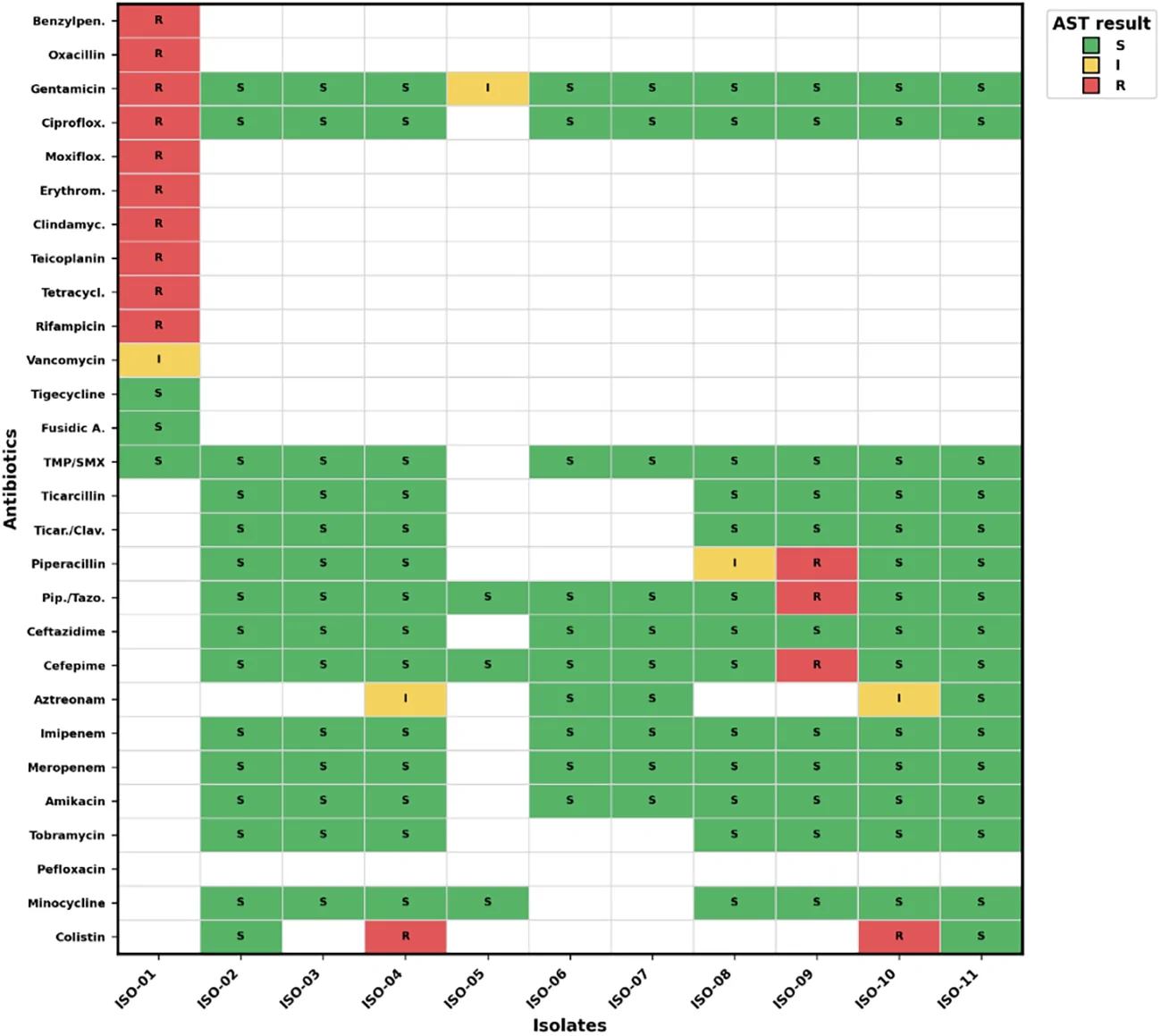
Heatmap of antimicrobial susceptibility patterns among bacterial isolates.

#### 3.3.2 Antibiotic susceptibility for Gram-negative bacteria

In **Tables 4** and **5**, the results showed that *P. alcaligenes* was susceptible to all of the agents tested and *M. lacunata* was susceptible to all of the agents tested except colistin, to which it was resistant, while the two isolates of *A. sobria* are sensitive to piperacillin/tazobactam, ceftazidime, cefepime, aztreonam, imipenem, meropenem, gentamicin, ciprofloxacin and trimethoprim/sulfamethoxazole. Moreover, *F. tularensis* is sensitive to piperacillin/tazobactam, cefepime, and minocycline. *A. haemolyticus* is sensitive to ticarcillin, ticarcillin/clavulanic acid, piperacillin, piperacillin/tazobactam, ceftazidime, cefepime, imipenem, meropenem, amikacin, gentamicin, tobramycin, ciprofloxacin, minocycline, colistin, and trimethoprim/sulfamethoxazole, whereas the two isolates of *Pseudomonas stutzeri* are sensitive to ticarcillin, ticarcillin/clavulanic acid, ceftazidime, imipenem, meropenem, amikacin, gentamicin, tobramycin, ciprofloxacin, minocycline, and trimethoprim/sulfamethoxazole, although one of the two isolates is resistant to piperacillin, piperacillin/tazobactam, and cefepime. The two isolates of *Sphingomonas paucimobilis* are sensitive to all antibiotics except one, which is resistant to colistin.

**Table 4:** Species-level antibiotic resistance rates with 95% confidence intervals.

Species	Gram reaction	Source hospital	No. of isolates	No. of tested results	S	I	R	Resistance rate (%)	95% CI	Non-susceptible rate (%)
*Granulicatella elegans*	Gram-positive	Merjan Teaching Hospital	1	14	3	1	10	71.4	45.4 -88.3	78.6
*Acinetobacter haemolyticus*	Gram-negative	Merjan Teaching Hospital	1	15	15	0	0	0.0	0.0 -20.4	0.0
*Pseudomonas alcaligenes*	Gram-negative	Merjan Teaching Hospital	1	14	14	0	0	0.0	0.0 -21.5	0.0
*Moraxella lacunata*	Gram-negative	Al-Jiumhori Hospital	1	16	14	1	1	6.2	1.1 -28.3	12.5
*Francisella tularensis*	Gram-negative	Al-Jiumhori Hospital	1	4	3	1	0	0.0	0.0 -49.0	25.0
*Aeromonas sobria*	Gram-negative	Al-Jiumhori Hospital	2	20	20	0	0	0.0	0.0 -16.1	0.0
*Pseudomonas stutzeri*	Gram-negative	Turkish Hospital	2	28	24	1	3	10.7	3.7 -27.2	14.3
*Sphingomonas paucimobilis*	Gram-negative	Turkish Hospital	2	32	30	1	1	3.1	0.6 -15.7	6.2

**Table 5:** Pooled antibiotic resistance according to hospital source.

Hospital	No. of isolates	No. of tested results	S	I	R	Non-resistant (S+I)	Resistance rate (%)	95% CI
Merjan Teaching Hospital	3	43	32	1	10	33	23.3	13.2–37.7
Turkish Hospital	4	60	54	2	4	56	6.7	2.6–15.9
Al-Jiumhori Hospital	4	40	37	2	1	39	2.5	0.4–12.9

Recalculating the resistance rates (R-only) showed that *Granulicatella elegans* had the highest rate. *Pseudomonas stutzeri* and *Moraxella lacunata* were less resistant, and *Acinetobacter haemolyticus*, *Pseudomonas alcaligenes*, *Francisella tularensis*, and *Aeromonas sobria* were no longer resistant in the recalculated summary. All confidence intervals in **Table 4** were recomputed by the Wilson score method so that they are derived consistently with those reported in **Tables 3**, **5**, **7**, and 8; the intervals are correspondingly wide, as expected for the small number of results available per species.

The species-level resistance percentages are plotted in the vertical column chart with error bars. The error bars represent the recomputed Wilson score 95% confidence intervals given in **Table 4**, and the vertical axis has been extended to 100% so that the upper limits are fully displayed; the ranking of species is unchanged. *Granulicatella elegans* was the most highly resistant, followed by *Pseudomonas stutzeri* and *Moraxella lacunata*, while the other species exhibited low or no resistance (**Figure 4**).

**Figure 4:**
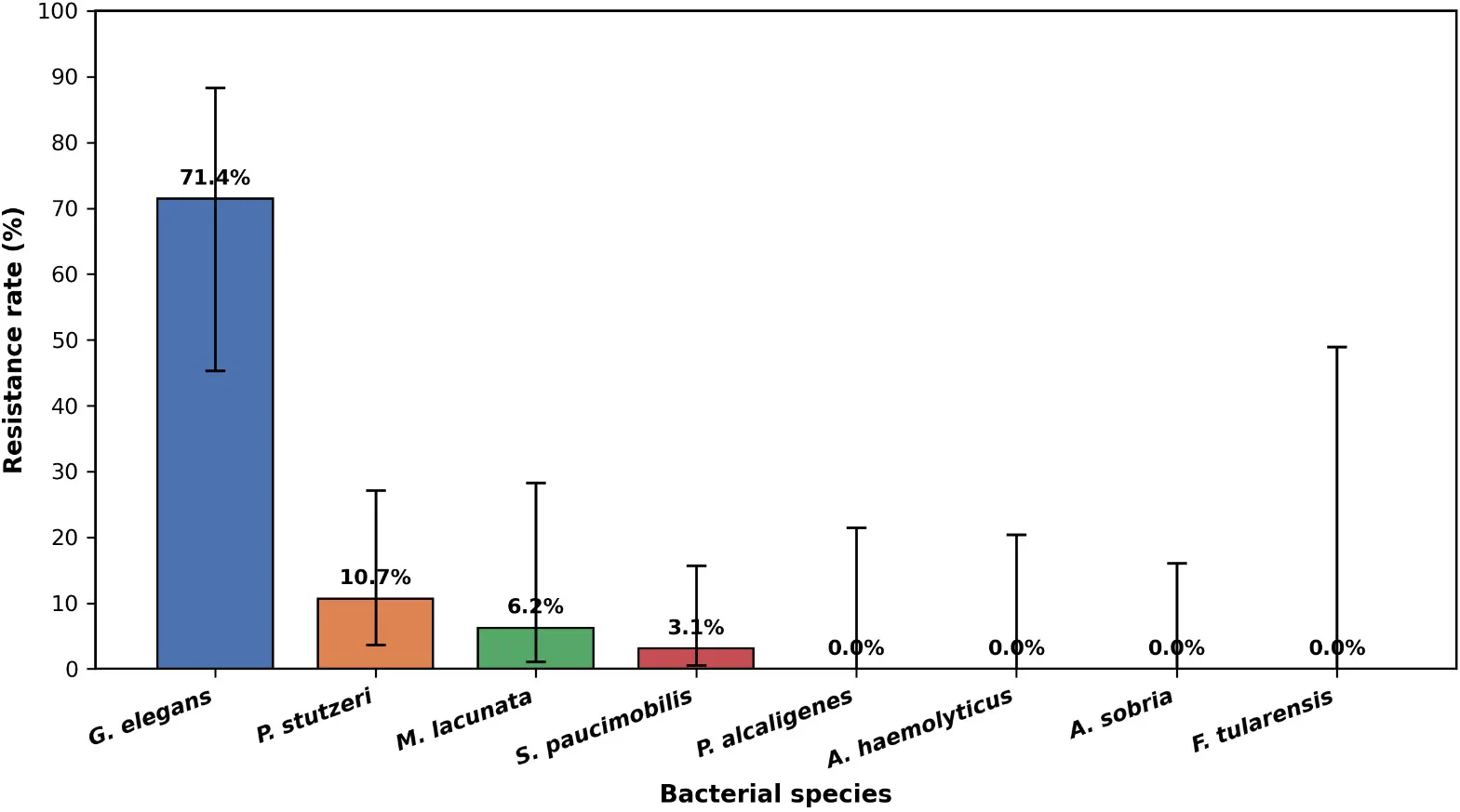
Antibiotic resistance rates with 95% confidence intervals among bacterial species.

The combined resistance rate was highest in Merjan Teaching Hospital (23.3%), mostly due to the predominance of most resistant results in the single Gram-positive *Granulicatella elegans* isolate. The pooled resistance of Turkish Hospital was 6.7% and of Al-Jiumhori Hospital was 2.5%. The *p*-value for exploratory chi-square comparison across the hospitals was 0.004, but exact pairwise tests are preferred due to small expected counts.

As shown in **Tables 4** and **5**, most Gram-negative isolates were fully susceptible (resistance rate 0%). The exceptions were *Moraxella lacunata*, which was resistant to colistin (species-level resistance rate, 6.2%), and *Pseudomonas stutzeri*, in which one of the two isolates was resistant to piperacillin, piperacillin/tazobactam, and cefepime (species-level resistance rate of 10.7% or 21.4% for the single resistant isolate). Overall, therefore, the single Gram-positive isolate exhibited markedly higher resistance than the Gram-negative isolates; however, as discussed below, this contrast rests on only one Gram-positive organism and should be regarded as a descriptive observation rather than a generalizable group difference.

The vertical column chart shows the antibiotics ranked by the number of resistant results. The antibiotic names were shortened, each antibiotic was color coded, and the numbers over the columns were shortened to percentages for quick and easy viewing (**Figure 5**).

**Figure 5:**
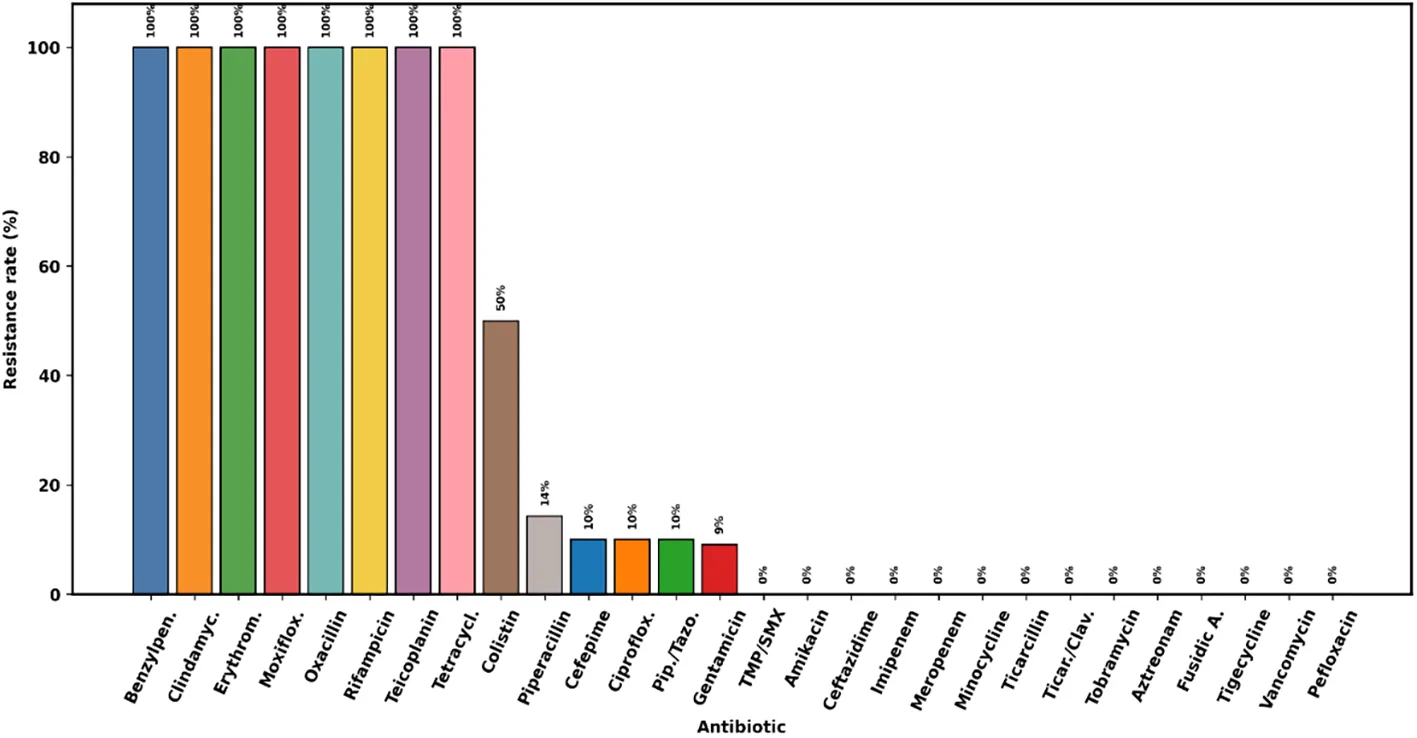
Frequency of antibiotic resistance among the tested antibiotics.

The pairwise exact testing indicated that the pooled resistance of samples from Merjan Teaching Hospital was statistically different from that of samples from the other hospitals before Bonferroni adjustment and still different from that of samples from Al-Jiumhori Hospital after Bonferroni adjustment. These comparisons are exploratory since the antibiotic results are aggregated across isolates and antibiotics (**Table 6**).

**Table 6:** Pairwise Fisher’s exact comparisons of pooled resistance between hospitals.

Pairwise comparison	Fisher’s exact *p*-value	Bonferroni-adjusted *p*-value
Merjan Teaching Hospital vs. Turkish Hospital	0.020	0.061
Merjan Teaching Hospital vs. Al-Jiumhori Hospital	0.008	0.023
Turkish Hospital vs. Al-Jiumhori Hospital	0.645	1.000

The Gram-positive isolate had a much higher pooled R-only resistance rate than the Gram-negative isolates (**Table 7**). This finding, however, is on the basis of only one Gram-positive isolate and should be considered an important descriptive observation rather than a generalizable group comparison.

**Table 7:** Pooled antibiotic resistance according to Gram reaction.

Gram reaction	No. of isolates	No. of tested results	S	I	R	Non-resistant (S+I)	Resistance rate (%)	95% CI	Fisher’s exact *p*-value
Gram-positive	1	14	3	1	10	4	71.4	45.4–88.3	<0.001
Gram-negative	10	129	120	4	5	124	3.9	1.7–8.8	<0.001

Antibiotic-wise analysis identifies the antibiotic(s) that led to the resistance profile (**Table 8**). With several Gram-positive antibiotics, 100% resistance was observed, which was due to testing against only one isolate and thus very wide confidence intervals. Colistin resistance was only detected in two out of four tested results; therefore, the estimate was high, but with a relatively low denominator.

**Table 8:** Antibiotic-wise resistance frequency among the tested bacterial isolates.

Antibiotic	No. of tested results	S	I	R	Resistance rate (%)	95% CI
Colistin	4	2	0	2	50.0	15.0–85.0
Tetracycline	1	0	0	1	100.0	20.7–100.0
Teicoplanin	1	0	0	1	100.0	20.7–100.0
Rifampicin	1	0	0	1	100.0	20.7–100.0
Oxacillin	1	0	0	1	100.0	20.7–100.0
Moxifloxacin	1	0	0	1	100.0	20.7–100.0
Erythromycin	1	0	0	1	100.0	20.7–100.0
Clindamycin	1	0	0	1	100.0	20.7–100.0
Benzylpenicillin	1	0	0	1	100.0	20.7–100.0
Piperacillin	7	5	1	1	14.3	2.6–51.3
Piperacillin/tazobactam	10	9	0	1	10.0	1.8–40.4
Ciprofloxacin	10	9	0	1	10.0	1.8–40.4
Cefepime	10	9	0	1	10.0	1.8–40.4
Gentamicin	11	9	1	1	9.1	1.6–37.7
Vancomycin	1	0	1	0	0.0	0.0–79.3
Trimethoprim/sulfamethoxazole	10	10	0	0	0.0	0.0–27.8
Tobramycin	7	7	0	0	0.0	0.0–35.4
Tigecycline	1	1	0	0	0.0	0.0–79.3
Ticarcillin/clavulanic acid	7	7	0	0	0.0	0.0–35.4
Ticarcillin	7	7	0	0	0.0	0.0–35.4
Minocycline	8	8	0	0	0.0	0.0–32.4
Meropenem	9	9	0	0	0.0	0.0–29.9
Imipenem	9	9	0	0	0.0	0.0–29.9
Fusidic acid	1	1	0	0	0.0	0.0–79.3
Ceftazidime	9	9	0	0	0.0	0.0–29.9
Aztreonam	5	3	2	0	0.0	0.0–43.4
Amikacin	9	9	0	0	0.0	0.0–29.9

Inconsistency check reveals that there are a couple of resistance percentages that need clarification (**Table 9**). The most relevant corrections are as follows: *Granulicatella elegans*—71.4% instead of 92.8%, *Pseudomonas stutzeri*—10.7% (combined results from two isolates) instead of 21.4% (single isolate), and *Sphingomonas paucimobilis*—one isolate with a colistin resistance result instead of no isolates.

**Table 9:** Data consistency check between the reported and the recalculated resistance percentages.

Item	Reported resistance, %	Recalculated R-only, %	Recalculated numerator/denominator	Comment
*Granulicatella elegans*	92.8	71.4	10/14	Reported 92.8% is not reproduced from R-only counts; R+I gives 11/14 = 78.6%
*Moraxella lacunata*	6.66	6.2	1/16	The raw matrix gives 1 resistant result among 16 tested results, equal to 6.25%
*Pseudomonas stutzeri*	21.4	10.7 species level; 21.4 for ISO-09	3/28 species level; 3/14 for ISO-09	The 21.4% value describes one resistant isolate only; the combined species-level rate across two isolates is 10.7%
*Sphingomonas paucimobilis*	0	3.1 species level; 6.2 for ISO-10	1/32 species level; 1/16 for ISO-10	The raw matrix records colistin resistance in ISO-10, so the value should be checked/corrected

## 4 Discussion

One of the most significant environments for bacteria on earth is water. Water is therefore another important medium for the spread of germs throughout various environmental compartments. The so-called urban water cycle, which includes drinking water, surface water, and garbage, was created by human activity and may involve the potential exchange of microorganisms. As a result, germs can spread from contaminated water environments (like wastewater) to pristine or pure water environments (like disinfected and spring drinking water) and ultimately make their way to people (Vaz-Moreira et al., 2014). The drinking water distribution system’s bacterial ecology was impacted by a number of environmental factors (Wang et al., 2018). Public health is significantly impacted by the microbiological quality of water in premise-plumbing systems, Microbial water quality is largely dependent on the structure of the bacterial community. The top five most prevalent phyla (*Proteobacteria*, *Actinobacteria*, *Bacteroidetes*, *Cyanobacteria*, and *Firmicutes*) have a combined relative abundance of over 90% out of the 24 phyla that were found. Mycobacteriaceae, unclassified Alphaproteobacteria, unclassified Corynebacteriales, Sphingomonadaceae, and Burkholderiaceae were the most prevalent families (Zhang et al., 2021). While 36.7% and 67.8% of the standard populations from sites inside the relevant distribution systems were MAR, the MAR standard bacteria were detected at frequencies of 20.4% and 18.6% in two different raw water supplies for two towns. According to isolate identification, MAR Gram-positive cocci and MAR Gram-negative, non-fermentative rods (*Pseudomonas*, *Alcaligenes*, *Moraxella*-like group M, and *Acinetobacter*) were more common in drinking water than in untreated source waters. The presence and the growth of microorganisms in drinking water distribution systems are routinely monitored in order to assess the biological stability of water without a residual disinfectant (Vavourakis et al., 2020). Drinking water distribution networks support vast microbiomes with different biofilm communities regardless of treatment, disinfection, or operational procedures. In Mediterranean areas, higher temperatures can promote reactions and microbiological growth that may raise esthetic water quality issues (Del Olmo et al., 2021). Waterborne illnesses, including diarrhea and emesis, have become more common as a result of consuming contaminated water. Inadequate treatment for certain waterborne illnesses can result in mortality. A significant public health problem has been ensuring the safety of drinking water quality. Risk in human health is posed by water contaminated with harmful microbes. It is vital to research about many water quality indicator systems that are employed globally and their effectiveness with the current water quality. Many microorganisms are utilized as major indicators to assess water quality, even though water quality regulations recommend using pathogenic microbes like enteric viruses as an indication (Wen et al., 2020).

The negative charge of the outer membrane and LPS of Gram-negative bacteria, as well as the resilience or tolerance of Gram-positive bacteria to high salt concentrations, may help to explain how the pathogen survives in highly contaminated water, particularly when using various antibiotics (Kafilzadeh et al., 2013). An important public health issue, the rising prevalence of bacterial infections that are extensively drug resistant (XDR) or multidrug resistant (MDR), puts a financial strain on the healthcare system because of longer hospital stays and increased morbidity and death (Ventola, 2015). In order to treat polymicrobial MDR infections, tigecycline, an antibacterial medication belonging to the tetracycline class, was developed (Tasina et al., 2011). There are organisms that are both Gram-positive and Gram-negative bacteria. Tygacil, or GAR-936, is the first parenteral semisynthetic drug in the glycylcycline class (Guay, 2004). It was approved by the Food and Drugs Administration in 2005 (Stein and Babinchak, 2013). The FDA warned in 2010 that using tigecycline to treat sepsis and severe infections was substantially linked to a higher risk of death from all causes (FDA, 2010). Tigecycline is a parenteral glycylcycline antibiotic that has bacteriostatic properties. It is structurally similar to tetracycline and has a fivefold higher binding affinity (Stein and Babinchak, 2013). As with other tetracycline, tigecycline binding limits peptide synthesis and bacterial growth by blocking the incorporation of amino acid residues, which hinders the elongation of peptide chains (Kaewpoowat and Ostrosky-Zeichner, 2015). Some pathogens are intrinsically resistant to tigecycline (Jenner et al., 2013), while numerous bacterial species, including *Acinetobacter baumannii*, *Klebsiella pneumoniae*, *Enterobacter* spp., and *Bacteroides fragilis*, have been reported to exhibit acquired resistance to tigecycline (Sun et al., 2013). Information on tigecycline resistance in Gram-positive bacteria is scarce. Overexpression of the efflux pump is involved in multi-antimicrobial extrusion. It was proposed that MepA reduces *Staphylococcus aureus*’ susceptibility to tigecycline, although it does not give resistance (Jenner et al., 2013). Historically known as nutritionally variable streptococci, *Abiotrophia* and *Granulicatella* species can cause endocarditis and other illnesses (Giuliano et al., 2012). Compared to endocarditis produced by streptococci, endocarditis induced by these organisms may be more severe and have a higher relapse rate, a longer course of illness, and a higher fatality rate (Padmaja et al., 2014). The American Heart Association recommends using penicillin (or ceftriaxone, if sensitive) to treat *Abiotrophia* and *Granulicatella* endocarditis (consequently, other severe infections brought on by organisms) (Baddour et al., 2015). Fusidic acid (FA) is a tetracyclic triterpene that was initially identified in 1960 in *Fusidium coccineum* (Godtfredsen et al., 1962). FA has been used in clinical settings since 1962 to treat both local and systemic staphylococcal infections, including coagulase-negative *Staphylococci* infections and methicillin-resistant *Staphylococcus aureus* (Zhao et al., 2013). Antibiotics with new modes of action and minimal drug resistance to bacteria are therefore required, as antibiotic resistance is a significant barrier to treating bacterial infections. The sole antibiotic that operates on this target is FA, which acts on EF-G (Fernandes, 2016). On the metabolism of FA have shown that the sodium salt of FA binds to protein strongly and reversibly and is well absorbed after oral treatment, with a bioavailability greater than 90% (Still et al., 2011). One of the primary adverse consequences of FA is hyperbilirubinemia, sometimes known as jaundice, due to its high protein binding rate (Lapham et al., 2016). A mixture of gentamycin and benzyl-penicillin has been utilized as an antibiotic treatment for infective endocarditis brought on by NVS (Cargill et al., 2012). When SMX is a structural counterpart of para-amino benzoic acid, it competes with it to stop dihydrofolic acid from being produced during an intermediate step of the biosynthesis of tetrahydrofolic acid (Brain et al., 2008). Trimetoprim, another bacteriostatic antibiotic, functions by inhibiting enzyme dihydrofolate reductase, which halts the synthesis of active metabolites of tetrahydrofolic acid (Masters et al., 2003). Because they can prevent and treat a lot of bacterial diseases, including urinary infections, both SMX and TRIM have been among the most commonly used antibiotics in human and veterinary medicine, aquaculture, and agriculture over the past 50 years (Carvalho and Santos, 2016). In order to affect DNA synthesis, SMX and TRIM progressively block the enzyme systems that bacteria use to produce tetrahydrofolic acid (Masters et al., 2003).

Diogo et al. (2024), in their study, gave significant details regarding the toxicity of trimethoprim and sulfamethoxazole to aquatic standard organisms. While TRIM was less hazardous but still had the potential to have a major impact at the biochemical, cellular, and individual levels, SMX was generally toxic or damaging, affecting multiple species and their physiological processes. Several publications reported the identification of SMX and TRIM in different environmental matrices around the world in recent years, with amounts ranging from nanogram to microgram per liter (e.g., wastewater, freshwater, marine, and groundwater) (Carvalho and Santos, 2016; Duan et al., 2022; Kovalakova et al., 2020). Carvalho and Santos (2016) considered that this increase is mostly brought on by direct water discharge. Antibiotics’ biological activity and ongoing entry into aquatic environments have been linked to an increase in concentrations, which has noticeable effects on non-target organisms at various trophic levels, such as bacteria (Diogo et al., 2023). The antibiotics can compromise several physiological and metabolic pathways and cause negative effects even at a low dosage (Rodrigues et al., 2016).

The antibiotics in the polymyxin family, which includes colistin (also known as polymyxin E) and polymyxin B, have had a unique destiny over the past 70 years. When the polymyxins were originally introduced to the market in the 1950s, they were hailed as “miracle” antibiotics because of their low level of resistance and bactericidal effectiveness against Gram-negative bacteria (Baron et al., 2016). In the 1950s, a number of research were able to understand the mechanism of action of colistin, but they were unable to fully explain it. The identification of *in vitro* resistant mutants at that time demonstrated polymyxin resistance (Olaitan et al., 2014). The intricacy of the processes by which bacteria protect themselves from colistin action was discovered through investigations into colistin resistance mechanisms (Olaitan et al., 2014). There may be more mechanisms underlying polymyxin resistance, as the initial targets found to be accountable for colistin resistance did not seem to be adequate to account for resistance in every isolate (Baron et al., 2016). In 1947, Koyama identified colistin as an ancient polypeptide antibiotic of group E in cultures of *Paenibacillus polymyxa* subspecies *colistinus* (Stansly and Schlosser, 1947). Although it is ineffective against Gram-positive bacteria, anaerobic bacteria, and mycoplasmas, it is a potent narrow-spectrum bactericidal agent against the majority of GNB (Poirel et al., 2017). The main target of polymyxins is the lipopolysaccharide of GNB membranes (Baron et al., 2016). The negatively charged lipid A of the outside portion of LPS interacts with divalent cations, primarily Mg^2+^ and Ca^2+^, to stabilize the outer membrane overall (Olaitan et al., 2014). As a result, the positively charged molecule colistin has a high affinity for binding to the LPS, which causes cations to be displaced by electrostatic contact. LPS is released as a result of the membrane structure being disorganized (Baron et al., 2016). Colistin’s lipophilic chain is subsequently used to insert it into the outer membrane. Colistin enables the outer membrane to insert itself and reach the inner membrane by changing its permeability. The integrity of the phospholipid bilayer is compromised, causing this inner membrane to become disorganized (Ahmed et al., 2020).

Multidrug-resistant bacteria are a serious global health hazard that has been brought about by the misuse and abuse of antibiotics (Nordmann and Poirel, 2016). Colistin is a last-line treatment for Gram-negative bacteria infections caused by multidrug-resistant and carbapenem-resistant bacteria (Liu et al., 2016). When this membrane eventually lyses, internal contents are released, and the bacteria die. Although the ultimate mechanisms causing cell death are still poorly understood, this process is the most widely used explanation to explain the antibacterial effect of colistin (Ahmed et al., 2020). Gram-negative bacteria known as *Moraxella* spp. can either be commensals or pathogens in animals, including pigs, sheep, cattle, and rabbits (Aebi, 2011). *Sphingomonas paucimobilis*, known as *Pseudomonas paucimobilis*, was identified as a human pathogen in 1979. According to phylogenetic evidence, it was renamed *S. paucimobilis* in 1990. As a waterborne organism, it is well known, and reports of diseases linked to tainted water sources have been made (Kallman et al., 2006). It has also been retrieved from a variety of sources inside the hospital setting, such as laboratory equipment, respiratory treatment equipment, and the hospital water system. There have been documented nosocomial epidemics of *S. paucimobilis*, which are thought to be caused by contaminated hospital equipment and surrounding (Meric et al., 2009). Environmental, non-fermenting, Gram-negative bacilli known as *Sphingomonas* species are known to be sporadic sources of primarily nosocomial illnesses (García-Lozano et al., 2015). *S. paucimobilis* is the primary cause of human illnesses; reports of other species are infrequent (Ionescu et al., 2022). A common non-fluorescent denitrifying bacteria in the environment, *Pseudomonas stutzeri*, was identified as an opportunistic pathogen in humans. Some strains can fix dinitrogen, while others interact with harmful metals or contribute to the breakdown of contaminants (Lalucat et al., 2006). In 1895, Burri and Stutzer published the first description of this bacteria. It is a non-fermenting, aerobic, Gram-negative bacillus. This complex include five different species: *P. stutzeri*, *P. xanthomarina*, *P. balearica*, *P. luteola*, and *P. chloritidismutans* (Li et al., 2022). Consequently, it was thought to be a low-virulence pathogen found in the environment, but over time it has come to be recognized as a source of infections in humans, especially in individuals who had risk factors or predisposing clinical conditions like immunosuppression (Lalucat et al., 2006).

The results of researchers Midhat and Abed (2023) showed that while the research isolates have shown sensitivity to the antibiotic tobramycin, *Pseudomonas* isolates exhibited strong resistance to piperacillin, gentamicin, and tetracycline, forming a high resistance rate of 100%. These findings were consistent with the current findings. The *Pseudomonas* bacteria are common pathogens that cause a significant number of infections with high rates of morbidity and mortality, particularly in patients who are hospitalized. *P. aeruginosa* is the most frequent cause of infections despite the fact that *Pseudomonas* has many species; infections caused by other *Pseudomonas* species are less common. The common Gram-negative bacterium *P. stutzeri* has been implicated in a number of diseases, especially in immunocompromised individuals; however, it has hardly ever been linked to infective endocarditis (Alwazzeh et al., 2020).

Interpreted in the context of the present study, the species recovered and their distribution across the three hospitals are consistent with a premise-plumbing origin dominated by non-fermentative and opportunistic Gram-negative bacteria. All isolates belonged to genera that are characteristically water- and biofilm-associated (*Pseudomonas*, *Acinetobacter*, *Moraxella*, *Aeromonas*, and *Sphingomonas*), supporting the interpretation that the contamination reflected colonization of the distribution and point-of-use plumbing rather than fecal pollution. The clustering of *Pseudomonas stutzeri* and *Sphingomonas paucimobilis* in Turkish Hospital, of *Aeromonas* in Al-Jiumhori Hospital, and of the single Gram-positive organism in Merjan Teaching Hospital suggests hospital-specific plumbing niches; however, because only one composite sample was examined per site, this pattern must be regarded as preliminary and hypothesis-generating rather than as evidence of genuine between-hospital differences.

With respect to antimicrobial resistance, the Gram-negative isolates in this study were largely susceptible, which is broadly consistent with reports of low to moderate resistance among environmental non-fermenters recovered from hospitals and drinking-water systems, including surveys that have traced Acinetobacter populations from the water source to the tap (Narciso-da-Rocha et al., 2013) and community analyses of premise-plumbing outlets (Zhang et al., 2021), although directly comparable Iraqi and regional data remain limited. The reduced susceptibility observed in *Pseudomonas stutzeri* to piperacillin, piperacillin/tazobactam, and cefepime and the colistin result in *Moraxella lacunata* are in line with the recognized capacity of environmental *Pseudomonas* and related genera to acquire or express resistance determinants, as previously described for water-associated *Pseudomonas* populations (Ono et al., 2023). By contrast, several earlier hospital water studies from Iraq and the wider Middle East have reported substantially higher multidrug resistance frequencies; the comparatively low rates found here most likely reflect the small number of isolates and the predominance of environmental rather than clinical strains and should not be taken to indicate an absence of resistance risk.

The single Gram-positive isolate, *Granulicatella elegans*, accounted for almost all of the resistant results and was the principal driver of the elevated pooled resistance recorded for Merjan Teaching Hospital. Several factors may explain this disproportionate resistance. As a fastidious, nutritionally variant organism, *G. elegans* is intrinsically less susceptible to a number of agents and is notoriously difficult to test reliably by automated systems so that some of the resistant calls may reflect a testing artefact as much as true resistance. In addition, the thick peptidoglycan cell wall of Gram-positive organisms, together with the absence of the outer membrane permeability barrier that in Gram-negative bacteria can both exclude many antibiotics and, for agents such as colistin, provide their target, means that the susceptibility panels applied to Gram-positive and Gram-negative isolates are not directly comparable. The apparent excess resistance of *G. elegans* therefore reflects a combination of intrinsic biology, panel composition, and the fact that the observation rests on a single isolate and is best interpreted as a descriptive finding rather than a generalizable group difference.

A specific note of caution applies to the isolate identified by VITEK 2 as *Francisella tularensis*. *F. tularensis* is the causative agent of tularaemia, a notifiable disease, and is classified as a category A select agent requiring biosafety level 3 containment; its recovery from routine hospital tap water on the basis of an automated phenotypic system alone would constitute an extraordinary claim. The VITEK 2 Gram-negative database is not validated for *Francisella* species, and fastidious coccobacilli are well recognized to be misidentified by automated biochemical platforms. Because no 16S rRNA gene sequencing or other molecular confirmation was carried out, this result should be regarded as a probable misidentification rather than a confirmed detection of *F. tularensis* and most plausibly represents a phenotypically similar environmental Gram-negative coccobacillus. Had such an organism genuinely been present, it would have carried major biosafety, clinical, and public health implications and would have mandated immediate molecular confirmation, reference-laboratory referral, and notification under appropriate containment. The same caveat applies, to a lesser degree, to the other uncommon environmental species reported here (for example, *G. elegans* and *Aeromonas sobria*); accordingly, molecular confirmation of all isolates by 16S rRNA gene sequencing is identified as a priority for future work.

### 4.1 Limitations

This study has several limitations that should be considered when interpreting the findings. First, the survey was based on a single composite tap water sample from each of the three hospitals and had a total of only 11 isolates, with several species represented by a single isolate; the resistance percentages are therefore descriptive estimates with wide confidence intervals, and the apparent differences between hospitals, species, and Gram groups should be regarded as exploratory rather than definitive. Second, isolates were identified and tested using an automated phenotypic platform (VITEK 2) without molecular confirmation so that identifications of uncommon or fastidious organisms—most notably the presumptive *Francisella tularensis*—must be treated with caution and verified by 16S rRNA gene sequencing in future work. Third, antimicrobial susceptibility was determined as a single determination per isolate rather than in replicate, and the antibiotic panels differed between Gram-positive and Gram-negative organisms, which limits a direct comparison. Finally, the statistical comparisons were performed on aggregated antibiotic results with small expected counts and are presented for exploratory purposes only. Larger, multi-sample studies incorporating molecular identification and standardized, replicated susceptibility testing are required to confirm and extend these observations.

## Data Availability

The raw data supporting the conclusions of this article will be made available by the authors, without undue reservation.
